# Long non-coding RNA KCNQ1OT1 up-regulates CTNND1 by sponging miR-329-3p to induce the proliferation, migration, invasion, and inhibit apoptosis of colorectal cancer cells

**DOI:** 10.1186/s12935-020-01425-2

**Published:** 2020-07-24

**Authors:** Xing Liu, Yexiang Zhang, Yan Wang, Chao Bian, Fengji Wang

**Affiliations:** 1 Department of Anorectal Surgery, Jining NO. 1 People’s Hospital, Jining, 272000 Shandong China; 2 Department of Surgery, Second People’s Hospital, Rencheng District, Jining, 272061 Shandong China; 3 Department of Acupuncture and Physiotherapy, Jining NO. 1, People’s Hospital, Jining, 272000 Shandong China; 4 Department of General Surgery, Shandong Institute of Parasitic Diseases, Shandong First Medical University and Shandong Academy of Medical Sciences, No. 11 Taibaizhong Road, Jining, 272033 Shandong China

**Keywords:** Colorectal cancer, lncRNA KCNQ1OT1, miR-329-3p, CTNND1

## Abstract

**Background:**

Long non-coding RNAs (lncRNAs) have been certified to be involved in the occurrence and growth of diverse cancers, including CRC. The purpose of the research was to explore the effects of lncRNA KCNQ1 overlapping transcript 1 (KCNQ1OT1) on proliferation, migration, invasion, and apoptosis in CRC cells and its mechanism.

**Methods:**

The levels of KCNQ1OT1 and miR-329-3p were examined by quantitative real-time polymerase chain reaction (qRT-PCR) in CRC tissues and cells. The mRNA and protein levels of catenin delta-1 (CTNND1) were measured by qRT-PCR and western blot analysis, respectively. The targets of KCNQ1OT1 and miR-329-3p were predicted by online software and confirmed by luciferase reporter assay. The cell proliferation, migration, invasion, and apoptosis were examined using 3-(4, 5-dimethylthiazol-2-yl)-2, 5-diphenyltetrazolium bromide (MTT), transwell, and apoptosis assay. The expression levels of CyclinD1, Bcl-2, MMP9, Cleaved-casp-3, and E-cadherin in SW480 and LS1034 cells were gauged by western blot analysis. Xenograft tumor model was structured to prove the biological role of KCNQ1OT1 of CRC in vivo.

**Results:**

The levels of KCNQ1OT1 and CTNND1 were significantly increased in CRC tissues and cells. Knockdown of KCNQ1OT1 suppressed proliferation, migration, invasion, and induced apoptosis in CRC cells. Conversely, CTNND1 overexpression reversed the impact of KCNQ1OT1 knockdown on CRC cells. Moreover, CTNND1 was verified as a direct target of miR-329-3p, and miR-329-3p could specially bind to KCNQ1OT1. Also, the down-regulation of KCNQ1OT1 triggered the CRC progress by up-regulating CTNND1 expression in CRC cells. Besides, KCNQ1OT1 knockdown inhibited CRC tumor growth through the miR-329-3p/CTNND1 axis in vivo.

**Conclusion:**

Our results indicated that KCNQ1OT1 could positively regulate CTNND1 expression by sponging miR-329-3p, thereby boosting the progression of CRC. Our findings provided the underlying therapy targets for CRC.

## Highlights

1. The targeting relationship between miR-329-3p and CTNND1 was first verified.
2. The targeting relationship between miR-329-3p and KCNQ1OT1 was first verified.
3. The KCNQ1OT1/miR-329-3p/CTNND1 axis relationship was first verified.

### Background

Colorectal cancer (CRC), as the most popular cancer around the world, is seriously endangering the health of all humankind. Its incidence rate ranks second in men and third in women [1]. According to cancer statistics in the United States, about 147,950 people were diagnosed with CRC and nearly 53,200 people died of CRC in 2020 [2].

With the rapid increase in incidence, it is estimated that it exceeded 2.2 million new patients and 1.1 million deaths of CRC patients worldwide by 2030 [3]. CRC is becoming a more and more dangerous factor threatening public health. Since most CRC patients are already in the advanced stages when they receive drug treatment in the presence of clinical symptoms, and the majority of patients in the advanced stage can only rely on surgical treatment, with poor prognosis. Currently, some literature shows that different strategies have been used to treat various types of cancer in preclinical models [4–8]. For example, Singh et al. reported that Resistin, a pro-inflammatory cytokine, blocked G1 in colon cancer cells by up-regulating SOCS3 [9]. Muhammad et al. confirmed that Bitter melon extract repressed breast cancer growth in the preclinical model by inducing autophagic cell death [10]. In recent years, scholars have actively explored the relevant factors and pathogenesis of CRC, hoping to find effective methods for early prevention, early diagnosis, and early treatment.

Long noncoding RNAs (lncRNAs) were discovered in recent years with the development of bioinformatics. They play inhibitory or carcinogenic roles in the occurrence and development of various cancers. Prensner et al. pointed out that lncRNA-mediated biology holds a core position in cancer progression [11]. The expression of lncRNAs in cancer is generally out of balance, abnormal expression of lncRNAs activates the occurrence of cancer by disrupting normal cellular processes, usually by promoting epigenetic inhibition of downstream target genes [12]. LncRNA KCNQ1 overlapping transcript 1 (KCNQ1OT1), a member of the lncRNA family, is located at 11p15.5 with a full length of 91 kb. It is reported that KCNQ1OT1 is involved in the occurrence of various cancers, and serves as a carcinogen in CRC [13, 14]. However, the consequences are only part of many mechanisms of KCNQ1OT1 affecting CRC, and the exact mechanisms are fuzzy.

MicroRNAs (MiRNAs), a kind of highly conservative non-coding RNA with 19–22 nucleotides, could control the expression of post-transcriptional genes via binding to the 3′-untranslated region (3′-UTR) of the target mRNAs [15]. Some studies have demonstrated that miRNAs could be involved in the development and progression of various cancers. For example, the downregulation of miR-203 could repress ER-positive breast cancer growth and stemness by interacting with SOCS3 [16]. Also, miR-133b served as a tumor suppressor by inhibiting cell viability and migration in cervical cancer development [17]. MiRNAs have always been in the abnormal regulation state in tumor evolution, and participate in the nosogenesis of tumor along with lncRNAs [18]. Some exceptionally miRNAs have been demonstrated to regulate proliferation and apoptosis in CRC cells [19]. MiR-329-3p is situated in 4q32.31 and can be surveyed in bladder cancer [20], Cholangiocarcinoma [21], cervical cancer [22], and so on. The previous study has demonstrated that the expression of miR-329-3p was decreased in CRC [23]. However, the biological functions and relevant molecular mechanisms of miR-329-3p in CRC still need to be elucidated.

Catenin delta-1 (CTNND1), also called p120-catenin, was initially identified as the substrate Src of carcinogenic tyrosine kinase [24]. It is worth noting that CTNND1 is a multidimensional intracellular signal protein. According to its subcellular localization and E-cadherin expression, it can be used as both tumor inhibitor and metastasis promoter [25]. Thus, CTNND1 plays a critical role in tumor progression.

In our present research, we explored the regulatory role of KCNQ1OT1 in the proliferation, migration, invasion and apoptosis of CRC cells. And rescue experiments identified that KCNQ1OT1 contributed to CRC progression by regulating CTNND1 expression through binding to miR-329-3p. Furthermore, the effects of KCNQ1OT1 knockdown on tumor growth were further revealed in vivo.

## Materials and methods

### Clinical tissues and cell lines

Tumor tissues and paracancerous tissues of 30 CRC patients after operation were collected from Jining NO. 1 People’s Hospital. This study was approved by the ethics committee of Jining NO. 1 People’s Hospital, and all patients received no therapies before the operation and signed informed consent forms. These tissues were speedily frozen in liquid nitrogen for later use.

Normal human colon epithelial NCM460 cells and CRC cell lines (T84, LS1034, HCT116, and SW480) were obtained from the Shanghai Academy of Life Sciences (Shanghai, China). The NCM460, T84, and LS1034 cells were maintained in DMEM (Invitrogen, Carlsbad, CA, USA) with 10% FBS (Carlsbad), while the HCT116 and SW480 cells were cultured in RPMI 1640 (Carlsbad), 100 U/mL penicillin and 0.1 mg/mL streptomycin were added to all solutions. All cells were kept in an incubator with 5% CO_2_ at 37 °C.

### RNA extraction and RT-PCR

Trizol reagent (Invitrogen) was used to extract total RNA from cells and tissues, the total RNA was synthesized into cDNA by prime script ™ RT Reagent kit (TaKaRa, Dalian, China). And RT-PCR reaction was started on a 7300 machine (Thermo Fisher, Waltham, MA, USA) with the SYBR RT-PCR Kit (TaKaRa). The primer sequences presented in this study were as follows: KCNQ10T1 forward, 5′-CTTTGCAGCAACCTCCTTGT-3′ and reverse, 5′-TGGGGTGAGGGATCTGAA-3′; miR-329-3p forward, 5′-GGGAACACACCTGGTTAAC-3′ and reverse, 5′-CAGTGCGTGTCGTGGAGT-3′; CTNND1 forward, 5′-ATGTTTGCGAGGAAGCCGC-3′ and reverse, 5′-CGAGTGGTCCCATCATCTG-3′; GAPDH forward, 5′-GGAGCGAGATCCCTCCAAAAT-3′ and reverse, 5′-GGCTGTTGTCATACTTCTCATGG-3′; U6 forward, 5′-CAGCACATATACTAAAATTGGAACG-3′, and reverse, 5′-ACGAATTTGCGTGTCATCC-3′. GAPDH and U6 GAPDH were used to normalize the expression of a relative gene, and the 2^−ΔΔCt^ method was applied to compute the RNA level.

### Cell transfection

Small interfering RNAs specific to KCNQ10T1 (termed si-KCNQ10T1#1/2) and negative control siRNA (si-con) were synthesized and purchased from RiboBio Company (Guangzhou, China). MiR-329-3p mimic, miR-con, miR-329-3p inhibitor (anti-miR-329-3p), and anti-miR-con were obtained from RiboBio (Guangzhou, China). Moreover, to over-express CTNND1, the sequence of CTNND1 was cloned into a pcDNA3.1 empty vector (Invitrogen), named as pcDNA-CTNND1, and empty vector was considered as a negative control (pcDNA). According to the manufacturer’s instructions, all these oligonucleotides and plasmids were transfected into SW480 and LS1034 cells by using Lipofectamine 3000 reagent (Invitrogen).

### MTT assay

Cell proliferation was detected by MTT assay. SW480 and LS1034 cells were inoculated in the 96-well plates, respectively, cultured with the medium for 24 h, 48 h, 72 h, the MTT fluid (Beyotime, Haimen, China) was added and maintained for 4 h at room temperature. The supernatant was discarded, and DMSO was added to dissolve the formazan crystals. The microplate reader (Nanjing Detie Laboratory Equipment Co Ltd, Nanjing, China) was used to detect the optical density (OD) of each well at 490 nm.

### Flow cytometry assay

Approximately 4 × 10^3^ of SW480 and LS1034 cells were gathered and washed by PBS (Beyotime) twice. Afterward, following the manufacturer’s instructions 10 μL Annexin V-FITC (Carlsbad) and 5 μL PI staining solution (Carlsbad) were added to SW480 and LS1034 cells and kept in the dark at room temperature for 15 min. The Flow cytometry results were analyzed by FACScan flow cytometer (Treestar, Inc., San Carlos, CA, USA).

### Transwell assay

The invasion and migration of SW480 and LS1034 cells were explored by transwell experiments using transwell chambers (24-well plate, 8.0 μm; BD Biosciences, Franklin Lakes, NJ, USA). Invasion occurred when the chamber coated with matrigel (BD Biosciences), and it also could be used to test the migration without matrigel. SW480 and LS1034 cells were harvested and resuspended in medium without FBS after 48 h of transfection, 200 μL of cell suspension (1 × 10^5^/well) was added to each well in the upper chamber, and the lower chambers were covered with 10% FBS medium (600 μL/well), cultured at 37 °C for 24 h. Take out the upper chamber, treated it with 4% neutral formaldehyde for 10 min, dyed it with Giemsa for 10 min, and observed the number of cells passing through the membrane under an inverted microscope (Olympus, Tokyo, Japan).

### Western blot assay

The cell protein was extracted with RIPA reagent (Beyotime), and protein concentration was assessed with BCA reagent (Beyotime). After the protein was separated by 10% sodium dodecyl sulfate–polyacrylamide gel electrophoresis (SDS-PAGE), the protein was transferred to nitrocellulose membranes (Millipore, Billerica, MA, USA), and sealed in 5% skimmed milk for 1 h. And then, the membranes were overnight at 4 °C and incubated with GAPDH (1:5000, Abcam, Cambridge, UK), E-cadherin (1:1000; Abcam), CyclinD1 (1:1000; Abcam), Cleaved-casp-3 (1:1000; Abcam), Bcl-2 (1:1000; Abcam), MMP9 (1:1000; Abcam), E-cadherin (1:1000; Abcam). After the membranes were treated with the second antibody (Abcam), the protein bands were observed by ECL assay (Millipore).

### Dual-luciferase reporter assay

The KCNQ1OT1 wild type containing the predicted miR-329-3p binding sites or mutant type constructed by Sangon Biotech (Shanghai, China) were amplified and cloned into the pGL3 vectors (Promega, Madison, WI, USA), the vectors were transfected into SW480 and LS1034 cells interacted with miR-329-3p mimics or control. Afterward, pGL3-WT-CTNND1 or -MUT-CTNND1 was transfected in the same way. After 48 h a dual-luciferase reporting kit (Promega) was used to detect luciferase activities.

### In vivo experiment

This animal experiment was approved by the Animal Care and Use Committee of Jining NO. 1 People’s Hospital. First of all, the lentiviral vector (lenti-short hairpin, sh-KCNQ1OT1) for stable expression ofKCNQ1OT1 knockdown and its control (sh-con) were provided by Genechem (Shanghai, China). Moreover, BALB/c nude mice (4–5 weeks old) were purchased from Shanghai Animal Laboratory Center (Shanghai, China), and then mice were randomly divided into two groups (n = 5 per group). Subsequently, 1 × 10^7^ SW480 cells transfected with sh-KCNQ1OT1 or sh-con were subcutaneously injected the left flank of the nude mice. After 7 days of injection, tumor volume was tested every 7 days. Meanwhile, tumors were excised and weighed on day 35 after inoculation. Besides, qRT-PCR and western blot assays were applied to detect the levels of KCNQ1OT1, miR-329-3p, CTNND1, CyclinD1, Cleaved-casp-3, and Bcl-2 in xenograft.

### Statistical analysis

The differences of groups were analyzed by Student’s *t*-test and one-way analysis of variance (ANOVA), the Kaplan–Meier survival curves and the log-rank test were used to evaluate the survival of CRC patients, all the data was calculated by spss 22.0, the difference was considered to have statistically significant if *P *< 0.05.

## Results

### KCNQ1OT1 expression was notably up-regulated in CRC tissues and cells and was positively associated with poor prognosis of CRC patients

**Fig. 1:**
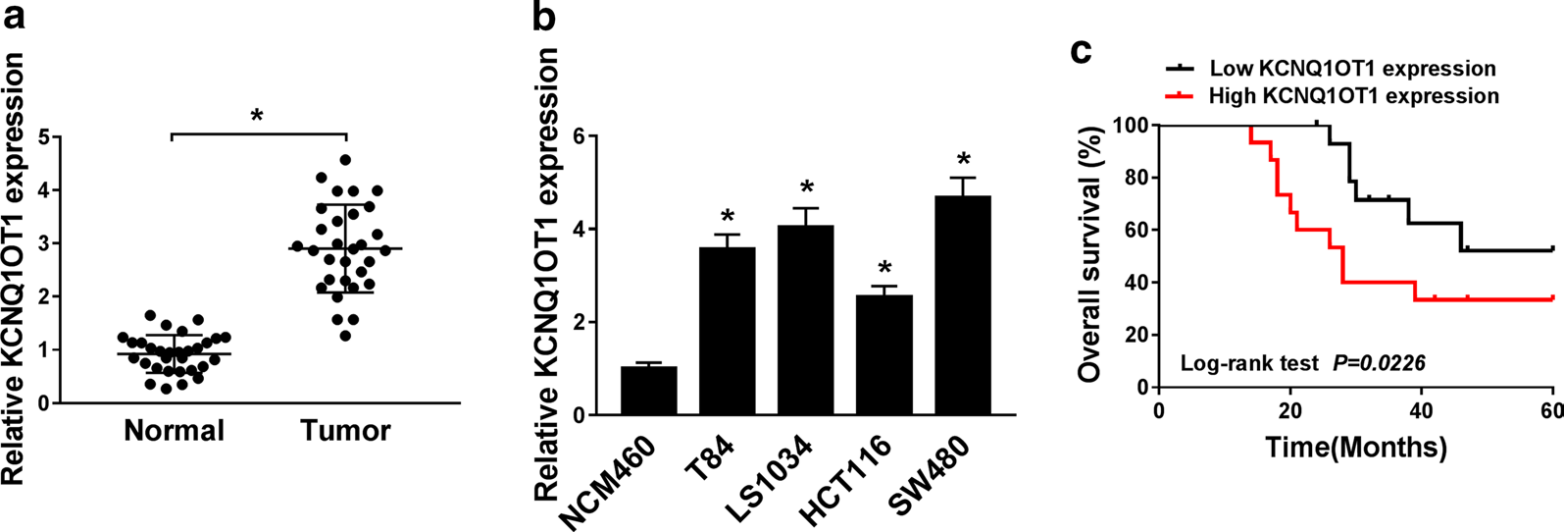
High expression of KCNQ1OT1 was a poor prognostic factor for CRC patients. **a**, **b** The expression level of KCNQ1OT1 in CRC tissues and cells was analyzed by qRT-PCR. **c** The correlation between KCNQ1OT1 expression and overall survival of CRC patients was analyzed with Kaplan–Meier method and log-rank test. *P < 0.05, vs. control group

At first, qRT-PCR assay was used to investigate the expression pattern of KCNQ1OT1 in CRC tissues and cells. Results exhibited that KCNQ1OT1 expression was remarkably increased in CRC tissues (n = 30) compared with adjacent normal tissues (n = 30) (*P *< 0.05, Fig. 1a). Compared with normal human colon epithelial cell line (NCM460), KCNQ1OT1 was highly expressed in CRC cell lines (T84, LS1034, HTC116, and SW480), especially in SW480 and LS1034 cells (*P *< 0.05, Fig. 1b). The patients were divided into a high expression group and a low expression group according to the median value of KCNQ1OT1 expression level, and Kaplan–Meier survival analysis implicated that CRC patients with higher KCNQ1OT1 expression have low survival rate and survival time (*P *= 0.0226, Fig. 1c). Meanwhile, the TCGA database also presented that the expression level of KCNQ1OT1 was inversely associated with the overall survival in CRC patients (*P *= 0.07, Additional file 1: Figure S1). Therefore, KCNQ1OT1 was considered as a factor with poor prognosis.

### KCNQ1OT1 knockdown inhibited cell proliferation, migration, invasion and induced apoptosis in CRC cells

**Fig. 2:**
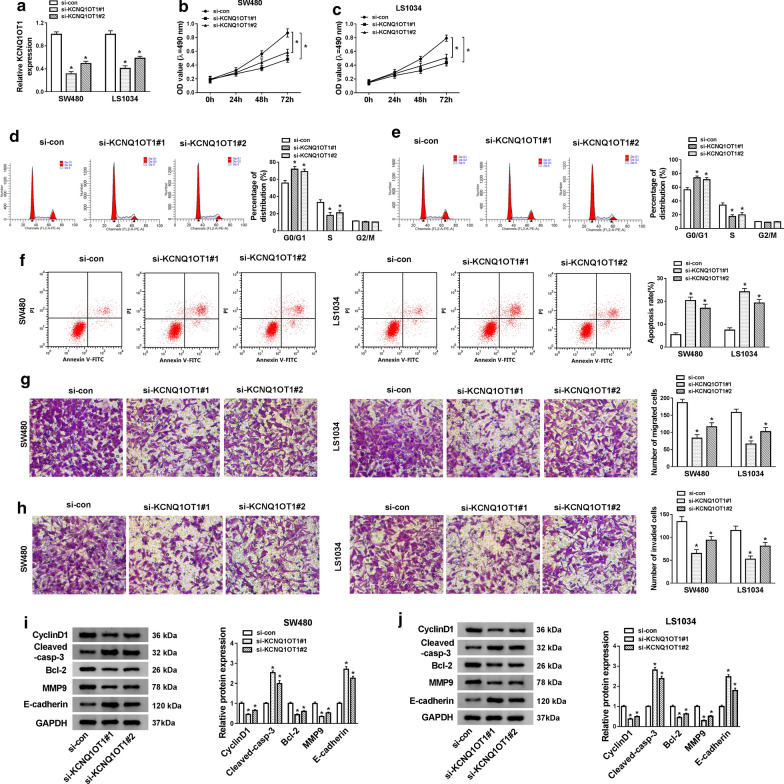
Knockdown of KCNQ1OT1 suppressed CRC cell proliferation, migration, invasion and promoted cell apoptosis. **a** The expression of KCNQ1OT1 was detected by qRT-PCR in SW480 and LS1034 cells by transfecting with specific siRNAs. **b**, **c** Proliferative ability of SW480 and LS1034 cells transfected with si-KCNQ1OT1#1 or si-KCNQ1OT1#2 was examined by MTT assay. **d**, **e** The cell cycle analysis of SW480 and LS1034 cells was detected by flow cytometry. **f** The cellular apoptosis of SW480 and LS1034 cells was tested by flow cytometry. **g**, **h** The migration and invasion of SW480 and LS1034 cells after transfected with si-KCNQ1OT1 were revealed by Transwell assays. **i, j** The protein levels of EMT markers (E-cadherin, MMP9), CyclinD1, apoptosis-associated proteins (Bcl-2, Cleaved-casp-3) were measured by western blot assays. *P < 0.05, vs. control group

After the SW480 and LS1034 cells were transfected with si-KCNQ1OT1#1, si-KCNQ1OT1#2, and si-con, qRT-PCR assay was applied to examine the expression level of KCNQ1OT1 in CRC in vitro. Result showed that KCNQ1OT1 expression was lower in si-KCNQ1OT1 (#1/2) group than si-con group (*P *< 0.05, Fig. 2a). To identify the function of KCNQ1OT1 on cells proliferation, MTT assay was performed in SW480 and LS1034 cells, the results showed that the proliferation abilities of CRC cells were reduced by the transfection of si-KCNQ1OT1 (#1/2) (*P *< 0.05, Fig. 2b, c). Then, a cell cycle analysis was performed by using flow cytometry in SW480 and LS1034 cells. As shown in Fig. 2d, e, the proportion of cells in the G0/G1 phase in the si-KCNQ1OT1 (#1/2) group significantly increased, whereas the cells in S phase decreased (*P *< 0.05) both compared with the si-con group. No significant difference was detected between the si-NC group and the si-KCNQ1OT1 (#1/2) group in the percentage of cells at the G2/M phase. Next, Flow cytometry analysis revealed that transfection of si-KCNQ1OT1 (#1/2) led to an obvious increase of apoptosis rates in both SW480 and LS1034 cells (*P *< 0.05, Fig. 2f). Migration and invasion ability of SW480 and LS1034 cells were hindered by the knockdown of KCNQ1OT1 (*P *< 0.05, Fig. 2g, h). Western blot results suggested that protein expression of CyclinD1, Bcl-2, and MMP9 have become down-regulated, while the expression of E-cadherin and the Cleaved-casp-3 were up-regulated (*P *< 0.05, Fig. 2i, j). CyclinD1 has been pointed out to promote cell proliferation [26]. Bcl-2 is an anti-apoptosis protein, and Cleaved-casp-3 is active form of caspase-3 (the apoptosis co-ordination enzyme) [27, 28]. MMP9 plays an important role in promoting the degradation of the extracellular matrix, thereby determining the invasiveness [29]. E-cadherin promotes epithelial cell adhesion [30]. Hence, decreased CyclinD1, Bcl-2, MMP9, and increased Cleaved-casp-3 and E-cadherin indicated the promotion of apoptosis rate and the suppression of proliferation, migration, and invasion. All of the data suggested that the KCNQ1OT1 knockdown may prevent the growth of CRC. Since the effect of si-KCNQ1OT1#1 on CRC cells was significant relative to si-KCNQ1OT1#2, hence, si- KCNQ1OT1#1 was selected for subsequent assays.

### MiR-329-3p acted as a target of KCNQ1OT1

**Fig. 3:**
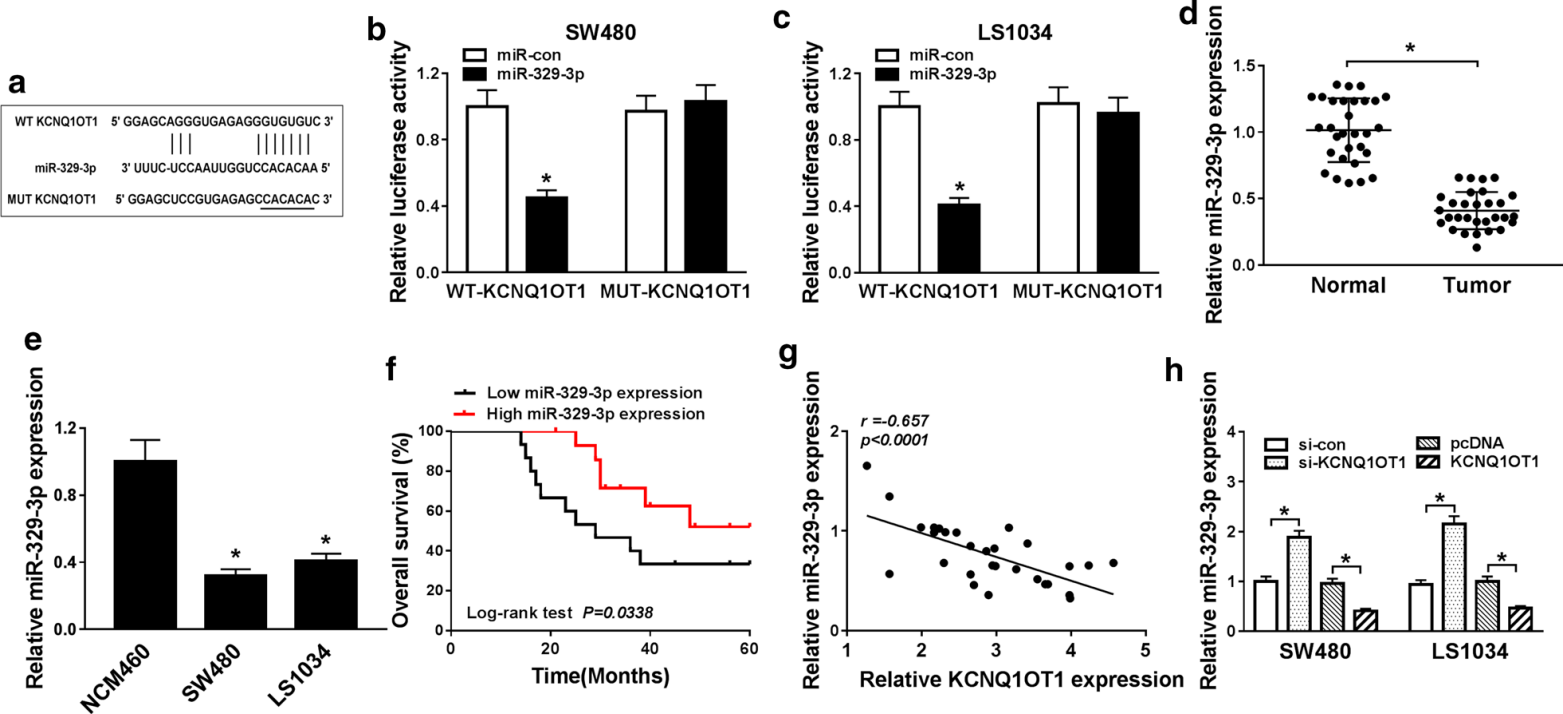
KCNQ1OT1 targeted miR-329-3p in CRC cells. **a** Predicted binding sites in KCNQ1OT1 and miR-329-3p were predicted by starBase v2.0. **b**, **c** Luciferase activity of SW480 and LS1034 cells was detected by dual-luciferase reporter assay. **d** The expression levels of miR-329-3p in CRC tissues and corresponding adjacent tissues were examined by qRT-PCR. **e** The expression level of miR-329-3p in CRC cells (LS1034, SW480) and normal cells (NCM460) was measured by qRT-PCR. **f** The correlation between miR-329-3p expression and overall survival of CRC patients was analyzed with Kaplan–Meier method. **g** The correlation between miR-329-3p and KCNQ1OT1 in human CRC samples was analyzed by Spearman’s correlation *(P *< 0.0001, r = − 0.657). **h** qRT-PCR was performed to measure miR-329-3p expression after transfected with KCNQ1OT1 or si-KCNQ1OT1 in SW480/LS1034 cells. *P < 0.05, vs. control group

KCNQ1OT1 was predicted to contain binding sites of miR-329-3p by bioinformatics software starBase v2.0 (Fig. 3a). A dual-luciferase reporter assay was performed to confirm the prediction. The results indicated that the luciferase activity of pMIR-REPOR-KCNQ1OT1-WT was dramatically reduced by the miR-329-3p mimic, but the pMIR-REPOR-KCNQ1OT1-MUT activity was not changed (*P *< 0.05, Fig. 3b, c). qRT-PCR analysis showed the expression of miR-329-3p was down-regulated in CRC tissues concerning with adjacent normal tissues (*P *< 0.05, Fig. 3d), as well as in CRC lines miR-329-3p expression was lower than that in normal colon cell (*P *< 0.05, Fig. 3e). Kaplan–Meier survival curves revealed that CRC patients with high miR-329-3p expression have better overall or disease-free survival than those with low miR-329-3p expression (*P *< 0.05, Fig. 3f). In our research, we found KCNQ1OT1 was negatively correlated with miR-329-3p in CRC (r = − 0.657, *P *< 0.0001, Fig. 3f). In SW480 and LS1034 cells, KCNQ1OT1 overexpression could inhibit miR-329-3p expression, whereas silencing KCNQ1OT1 could increase miR-329-3p expression (*P *< 0.05, Fig. 3h). These data indicated that miR-329-3p might act as a tumor suppressor in CRC.

### Silenced miR-329-3p expression reversed the partial function of si-KCNQ1OT1 on proliferation, migration, invasion, and apoptosis

**Fig. 4:**
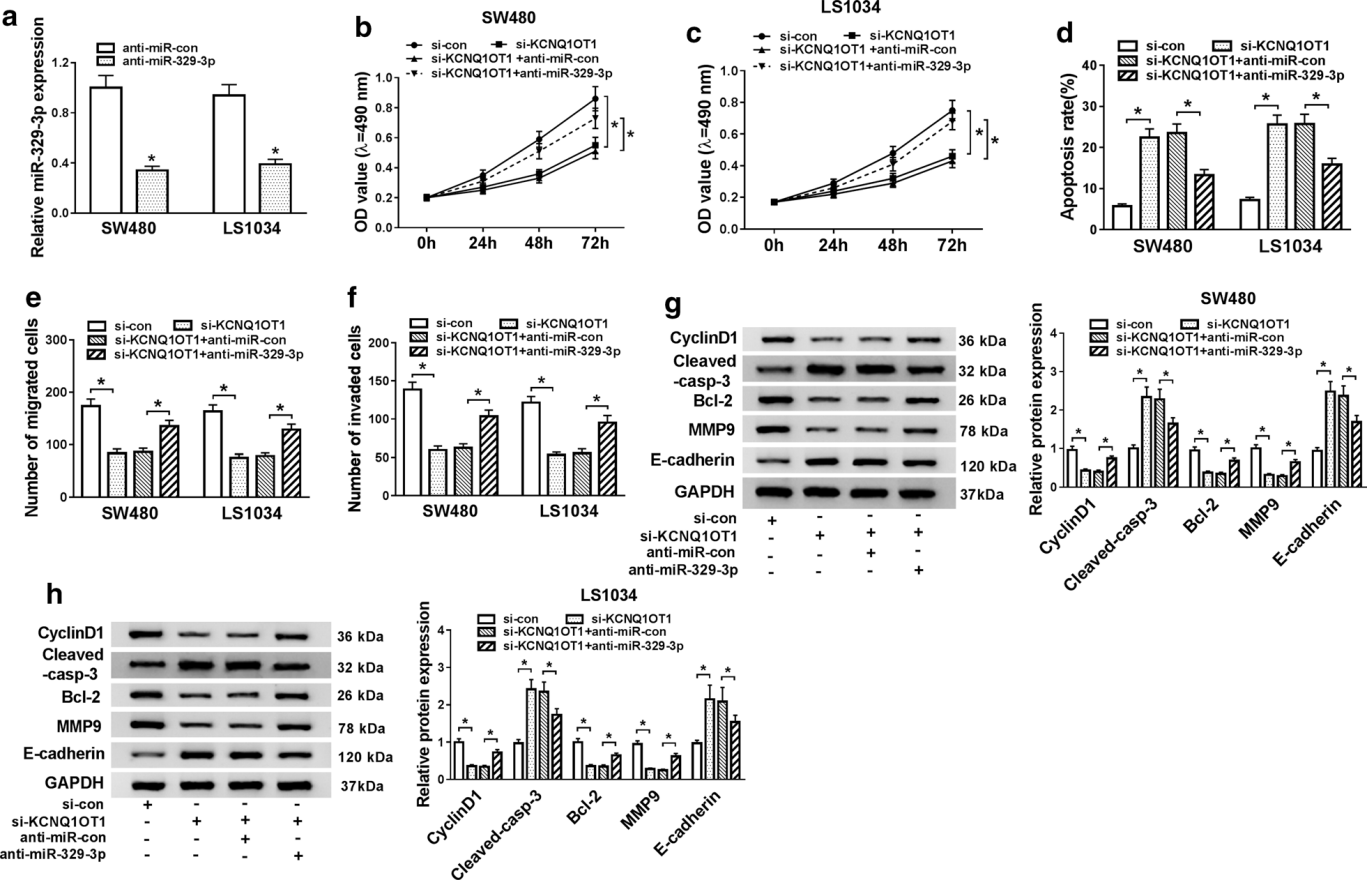
Silenced miR-329-3p expression reversed partial function of si-KCNQ1OT1 on proliferation, migration, invasion, and apoptosis. **a** The expression of miR-329-3p was detected by qRT-PCR after transfected with anti-miR-329-3p or anti-miR-329-3p-control (con) into SW480 and LS1034 cells. **b**, **c** The proliferative ability was measured after SW480 and LS1034 cells were transfected with si-KCNQ1OT1 or si-KCNQ1OT1 + anti-miR-329-3p. **d** Cell apoptosis was assessed by staining with annexin V and propidium iodide 48 h. the percentage of apoptotic cells was determined using flow cytometric analysis. **e**, **f** The cells migration and invasion were performed by MTT assay after transfected with si-KCNQ1OT1 or si-KCNQ1OT1 + anti-miR-329-3p. **g**, **h** The protein levels of EMT markers (E-cadherin, MMP9), CyclinD1, apoptosis-associated proteins (Bcl-2, Cleaved-casp-3) were measured in SW480 or LS1034 cells by western blot analysis. *P < 0.05, vs. control group

To further explore the relationship between KCNQ1OT1 and miR-329-3p in CRC cells, the mRNA level of miR-329-3p was measured in SW480 and LS1034 cells transfected with anti-miR-329-3p and anti-miR-NC, respectively. The results showed that lessened expression of miR-329-3p triggered a significant promotion in miR-329-3p expression in both SW480 and LS1034 cells (*P *< 0.05, Fig. 4a). Furthermore, cells were co-transfected with si-KCNQ1OT1 and anti-miR-329-3p to investigate their effects on cell proliferation, migration, invasion, and apoptosis by MTT assay, transwell assay, and flow cytometry, respectively. The results displayed that KCNQ1OT1 knockdown greatly constrained proliferation (*P *< 0.05, Fig. 4b, c), migration, invasion (*P *< 0.05, Fig. 4e, f), and enhanced apoptosis (*P *< 0.05, Fig. 4d) of SW480 and LS1034 cells, yet abnormally expression of miR-329-3p abrogated the effects. The expression levels of CyclinD1, Bcl-2, MMP9,Cleaved-casp-3, and E-cadherin proteins were detected by Western blot assay, the results demonstrated that after transfected with si-KCNQ1OT1 the levels of Bcl-2, MMP9,Cleaved-casp-3proteins were declined, while the levels of CyclinD1 and E-cadherin proteins were increased, whereas knockdown of miR-329-3p reversed the effects of KCNQ1OT1down-regulation on these proteins (*P *< 0.05, Fig. 4g, h).

### CTNND1 was a target of miR-329-3p in CRC cells

**Fig. 5:**
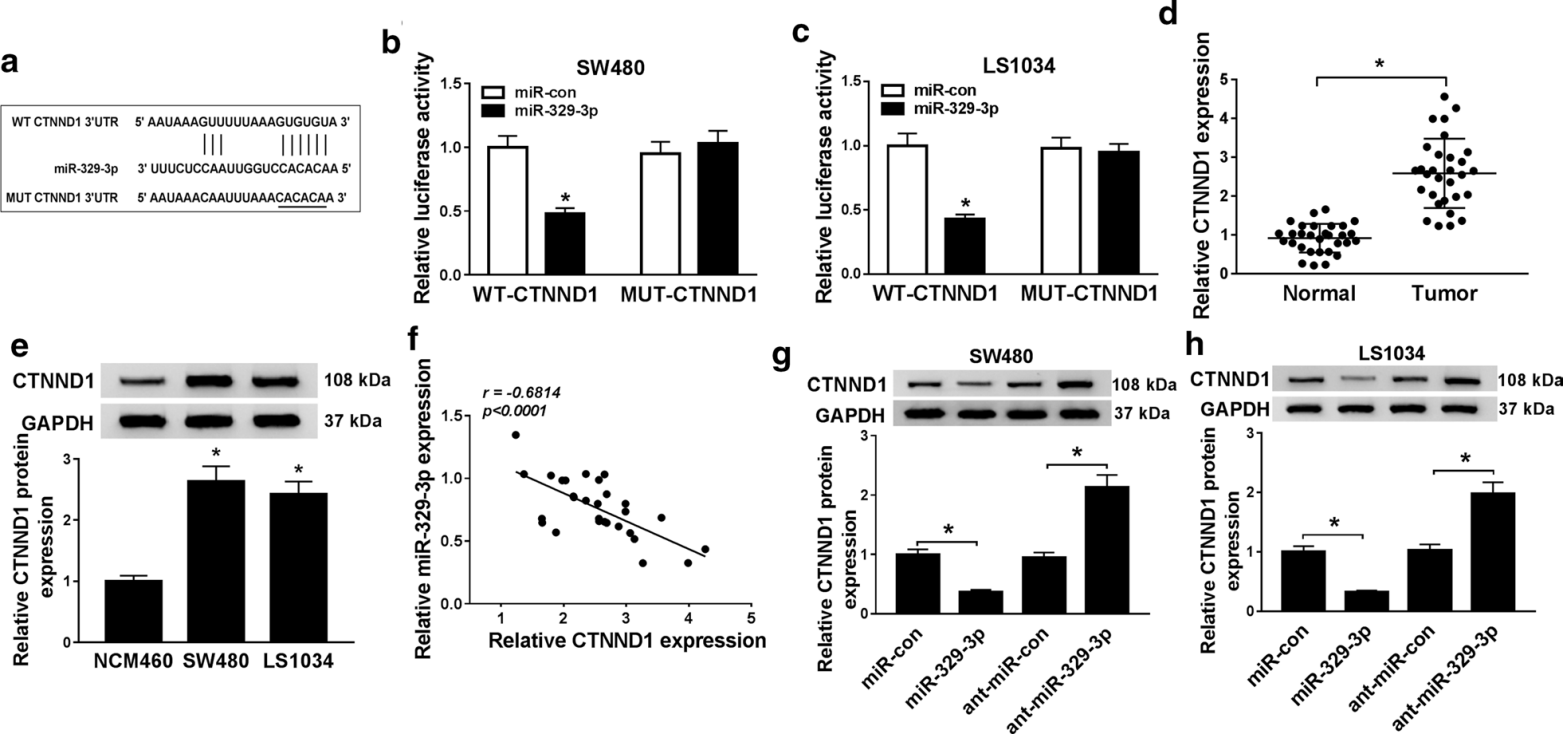
CTNND1 was a direct target gene of miR-329-3p. **a** The predicted binding sites of miR-329-3p and CTNND1. **b**, **c** Thedual-luciferase reporter assay was utilized to determine the luciferase activity of miR-329-3p and CTNND1. **d** The expression level of miR-329-3p in 30 CRC tissues and corresponding adjacent tissues was confirmed by qRT–PCR. **e** qRT-PCR was applied to determine the expression level of miR-329-3p in SW480 and LS1034 cells and normal colon NCM460 cell. **f** Spearman’s correlation analysis examined the correlations between the expression levels of CTNND1 mRNA and miR-329-3p in CRC tissues (*P *< 0.0001, r = − 0.6814). **g**, **h** The expression level of CTNND1 was measured by qRT-PCR after being transfected with miR-329-3p or anti-miR-329-3p for 48 h in SW480 and LS1034 cells. *P < 0.05, vs. control group

CTNND1 was a target of miR-329-3p was predicted by starBase v2.0, then pGL3 luciferase reporter plasmid containing the 3′-UTR sequences of wild type CTNND1 (CTNND1-WT) and mutant type CTNND1(CTNND1-MUT) were constructed (Fig. 5a). Dual-luciferase reporter assay was applied to validate the interaction between miR-329-3p and CTNND1, the luciferase activity of wild type CTNND1 (CTNND1-WT) was markedly decreased in SW480 and LS1034 cells, the luciferase activity of mutant type CTNND1 had no obvious change (*P *< 0.05, Fig. 5b, c). CTNND1 was highly expressed in CRC tissue samples and cells (*P *< 0.05, Fig. 5d, e). Spearman’s correlation analysis showed a negative correlation between CTNND1 and miR-329-3p (r =− 0.6814, *P *< 0.0001, Fig. 5d, e). The expression level of CTNND1 was detected in SW480 and LS1034 cells transfected with miR-329-3p and anti-miR-329-3p, respectively. The results confirmed that overexpression of miR-329-3p significantly reduced the expression levels of CTNND1, whereas knockdown of miR-329-3p significantly up-regulated the expression levels of CTNND1 in SW480 and LS1034 cells (*P *< 0.05, Fig. 5g, h). All data suggested that miR-329-3p targeted suppressed CTNND1 expression.

### CTNND1 overexpression reversed effects of miR-329-3p on proliferation, migration, invasion, and apoptosis of CRC cells

**Fig. 6:**
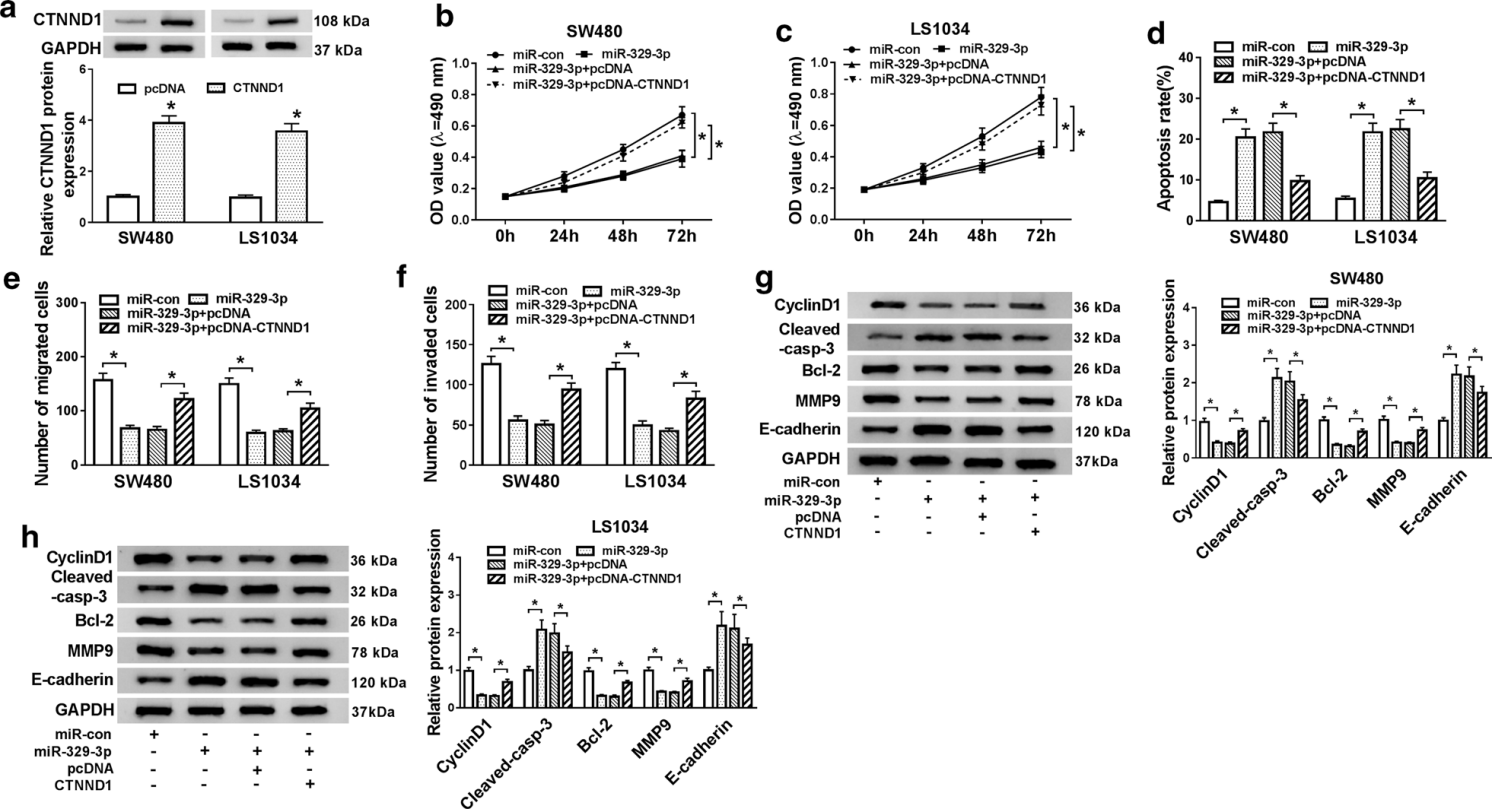
Overexpression of CTNND1 relieved the inhibitory effects of miR-329-3p on cell proliferation, migration, invasion and suppressed apoptosis. SW480 and LS1034 cells were transfected with control RNA or miR-329-3p together with plasmid expressing CTNND1 or empty vector. **a** The expression of CTNND1 in SW480 and LS1034 cells was tested by western blot. **b, c** SW480 and LS1034 cell proliferation were presented by MTT assay at 24 h, 48 h, and 72 h. **d** Flow cytometry was used to evaluate apoptosis of SW480 and LS1034 cells. **e**, **f** Cell migration and invasion in SW480 and LS1034 was detected by transwell assay. **g**, **h** The protein levels of EMT markers (E-cadherin, MMP9), CyclinD1, apoptosis-associated proteins (Bcl-2, Cleaved-casp-3) in SW480 and LS1034 cells were measured by western blot assays. *P < 0.05, vs. control group

The protein level of CTNND1 was measured in SW480 and LS1034 cells transfected with CTNND1 mimic and pcDNA, respectively. The results showed that enforced expression of CTNND1 triggered a significant promotion in CTNND1 expression in CRC cells (*P *< 0.05, Fig. 6a). Cells were co-transfected with miR-329-3p and pcDNA-CTNND1 to investigate their effects on cell proliferation, migration, invasion, and apoptosis. Data suggested that after CTNND1 highly expressed, the SW480 and LS1034 cells proliferation rate (*P *< 0.05, Fig. 6b, c), migratory and invasive ability were increased (*P *< 0.05, Fig. 6e, f), however, apoptosis ability was hindered (*P *< 0.05, Fig. 6d).These results indicate that CTNND1 may work as a tumor promoter in CRC cells. After SW480 and LS1034 cells were transfected with miR-329-3p mimic, we found the protein levels of CyclinD1, Bcl-2, MMP9 were decreased, while the protein levels of E-cadherin, Cleaved-casp-3 were increased, while these tendencies were overturned by co-transfection of pcDNA-CTNND1 (*P *< 0.05, Fig. 6g, h). We found that CTNND1 overexpression decreased the level of epithelial marker (E-cadherin), this phenomenon indicated that CTNND1 plays a key role in regulating EMT plasticity of CRC cells.

### KCNQ1OT1 positively modulated the expression of CTNND1 through sponging miR-329-3p

**Fig. 7:**
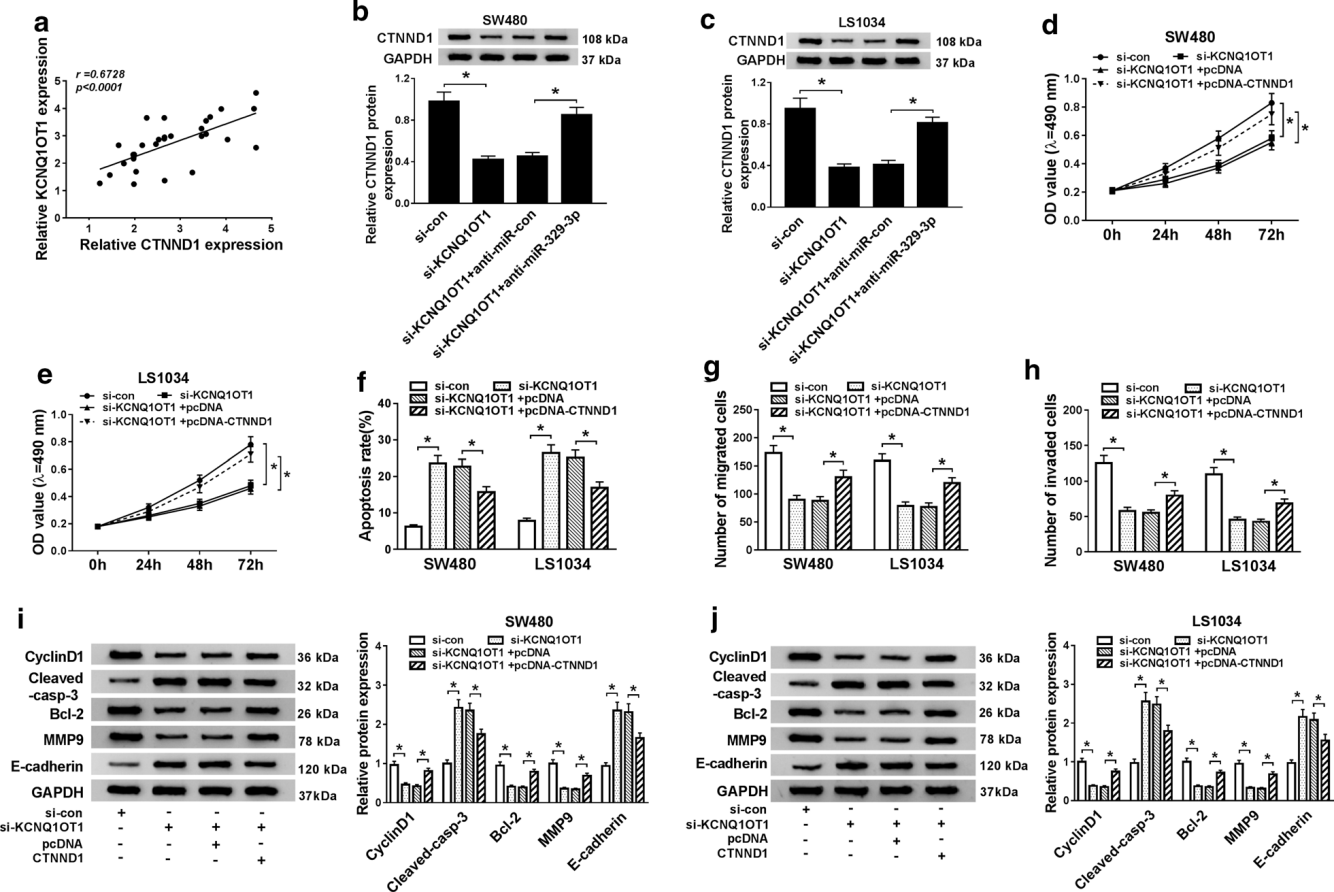
KCNQ1OT1 up-regulated CTNND1 expression via sponging miR-329-3p. **a** The correlation of KCNQ1OT1 and CTNND1 in CRC tissues was examined by spearman’s correlation analysis (*P *< 0.0001, r = − 0.6758). **b**, **c** SW480 and LS1034 cells were co-transfected with si-con/si-KCNQ1OT1 and si-KCNQ1OT1 + anti-con/si-KCNQ1OT1 + anti-miR-329-3p for 24 h, and then western blot was performed to check the level of CTNND1. **d**, **e** MTT assay was used to determine the viability of SW480 and LS1034 cells transfected with si-KCNQ1OT1 or si-KCNQ1OT1 + pcDNA-CTNND1. **f** And the cell apoptosis rates were measured by flow cytometry in SW480 and LS1034 cells transfected with si-KCNQ1OT1 or si-KCNQ1OT1 + pcDNA-CTNND1. **g**, **h** Transwell assay was conducted to evaluate the migratory and invasive ability of SW480 and LS1034 cells transfected with si-KCNQ1OT1 or si-KCNQ1OT1 + pcDNA-CTNND1. **i**, **j** The expression of EMT markers (E-cadherin, MMP9), CyclinD1, apoptosis-associated proteins (Bcl-2, Cleaved-casp-3) were evaluated by western blot in SW480 and LS1034 cells transfected with si-KCNQ1OT1 or si-KCNQ1OT1 + pcDNA-CTNND1. *P < 0.05, vs. control group

A similar positive correlation between KCNQ1OT1 and CTNND1 expression levels phenomenon was observed in CRC (r = 0.6728, *P *< 0.0001, Fig. 7a). The qRT-PCR results showed that si-KCNQ1OT1 could significantly reduce the CTNND1 expression, while CTNND1 expression was rescued after silencing miR-329-3p (*P *< 0.05, Fig. 7b, c). Base on those mentioned above, we predicted that miR-329-3p-mediated proliferative and metastatic inhibition was associated with CTNND1. Thus, we constructed CTNND1 over-expressing vector, and co-transfected into SW480 and LS1034 cells containing si-KCNQ1OT1, we found that reduced cell viability, migration, invasion, and increased apoptosis through si-KCNQ1OT1 mimic were overturned by the CTNND1 over-expression (*P *< 0.05, Fig. 7d–h). As well as, the CTNND1 over-expression reversed the decrease in protein levels of CyclinD1, Bcl-2, and MMP9, and the increase in protein levels of E-cadherin and Cleaved-casp-3 (*P *< 0.05, Fig. 7i, j). Taken together, all results suggested that KCNQ1OT1 regulated CTNND1 expression by sponging miR-329-3p.

### KCNQ1OT1 knockdown repressed CRC tumor growth in vivo

**Fig. 8:**
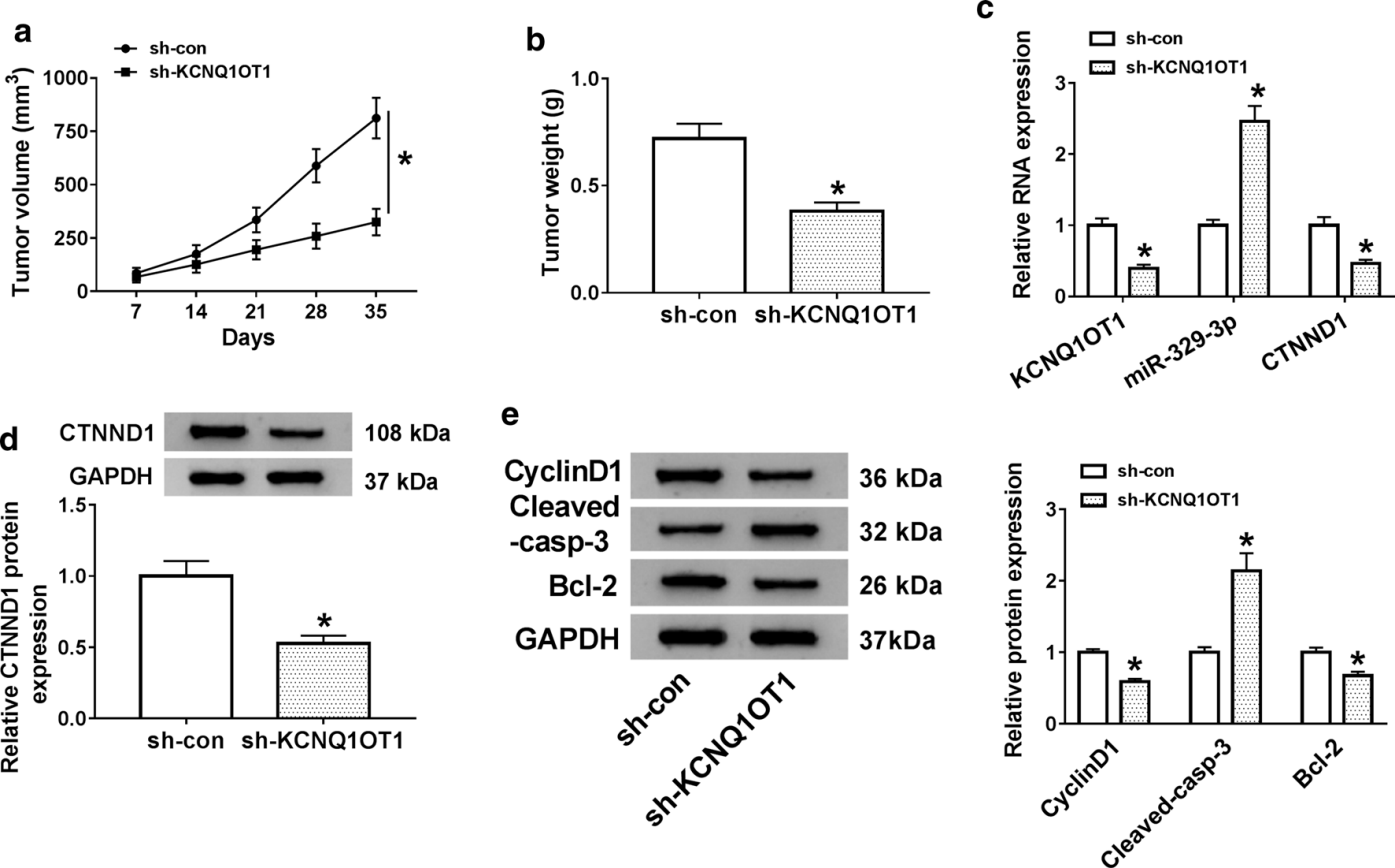
KCNQ1OT1knockdown suppressed CRC tumor growth by regulating of miR-329-3p/CTNND1axis in vivo. **a**, **b** Tumor volume and tumor weight were measured in xenografts. **c** The expression levels of KCNQ1OT1,miR-329-3p and CTNND1 in xenografts were assessed by RT-qPCR. **d** CTNND1 protein level was detected in xenografts. The protein levels of CyclinD1, Bcl-2, and Cleaved-casp-3 were measured in xenografts. **e** The expression levels of CyclinD1, apoptosis-associated proteins (Bcl-2, Cleaved-casp-3) and the control (GAPDH) were evaluated by western blot in xenografts transfected with sh-KCNQ1OT1 or sh-control. *P < 0.05, vs. control group

Finally, a mouse xenograft model of CRC was conducted to prove the functional role ofKCNQ1OT1 on tumor growth in vivo. As displayed in Fig. 8a, b, tumor volume and weight were reduced caused by KCNQ1OT1 downregulation, verifying that KCNQ1OT1 deficiency hindered tumor growth in vivo. Furthermore, qRT-PCR results suggested that the levels of KCNQ1OT1 and CTNND1 were declined in tumor tissues from the sh-KCNQ1OT1 group versus the sh-con group, while the miR-329-3p level was increased (Fig. 8c). Meanwhile, our data confirmed that the CTNND1 protein level was also decreased in xenograft (Fig. 8d). Then, in vivo assay, we verified that the silencing of KCNQ1OT1 led to a reduction in the protein levels of CyclinD1, Bcl-2, and enhancement in Cleaved-casp-3 protein level (Fig. 8e), suggesting that KCNQ1OT1 deficiency could repress proliferation and promote apoptosis in xenograft. That is to say, these findings implied that KCNQ1OT1deletion suppressed CRC tumor growth partly by regulating miR-329-3p/CTNND1 axis in vivo.

## Discussion

Colorectal cancer (CRC), as the most general gastrointestinal tumor occurring in the colorectal region, has an increasing incidence in China and the global. Although there have been some improvements in the diagnosis and treatment of CRC in recent years, the long-term survival rate has not been significantly improved, mainly due to the rapid proliferation of CRC and its relatively strong aggressive. Moreover, the processes of early diagnosis and treatment, standardized diagnosis and treatment are difficult to achieve. With the emergence of precision medicine, individualized diagnosis and therapies of patients have occupied dominant positions in the development of medicine in recent years. Therefore, the use of CRC biomarkers to improve the early diagnosis rate, evaluate the efficacy and prognosis of patients, explore effective individual clinical treatments, and monitor the patients’ conditions dynamically have become research hotspots to improve the prognosis of patients.

Recent studies have shown that changing the expression of lncRNAs contribute to the development and progression of human cancer and provide ideas for overcoming the problems of cancer therapy and drug resistance [31]. It has been indicated that lncRNA KCNQ1OT1 was up-regulated in CRC [14], according to the study, the level of KCNQ1OT1 in CRC tissues and cell lines were markedly increased relative to paired paracancerous tissues. These results suggested that KCNQ1OT1 may display a key role in the occurrence and progression. Besides, functional deficit experiments were applied to assess the potential impact of KCNQ1OT1 on CRC cells. The consequence showed that KCNQ1OT1 down-regulation could restrain the proliferation, migration, invasion, and enhance cell apoptosis in SW480 and LS1034 cells, which were matched with the consequences of Zhao et al. [32]. Also, CRC patients with low levels of KCNQ1OT1 have less chance of distant metastasis and effectively longer overall and disease-free survival. Hence, KCNQ1OT1 is a biomarker with a poor prognosis.

LncRNAs have been described as the function of internal miRNAs sponge, which combines with miRNAs and regulates their biological function [33, 34]. In our study, we found that KCNQ1OT1 included link sites for miR-329-3p. Numerous abnormally expressed miRNAs play significant roles in tumor evolution [35]. Accumulated researches showed that KCNQ1OT1 contributed to the chemo-resistance of CRC via sponging miR-34a, and KCNQ1OT1 expression was increased in MTX-resistant CRC and acted as the sponge of miR-760 [36]. But, the underlying molecular mechanism of KCNQ1OT1 associated with miR-329-3p in the course of CRC progression is misty. Our data confirmed that KCNQ1OT1 could act as a sponge of miR-329-3p to upregulate CTNND1 expression in vitro. Meanwhile, we conducted rescue assays to manifest that regulation of KCNQ1OT1 on proliferation, apoptosis, migration, and invasion was mediated by CTNND1, further validating that KCNQ1OT1 knockdown represses CRC progression via the miR-329-3p/CTNND1 axis in vitro. Apart from that, our data verified that KCNQ1OT1 deficiency could suppress CRC tumor growth partly through the miR-329-3p/CTNND1 pathway in vivo. Hence, it was predicted that KCNQ1OT1 utilized its function via the miR-329-3p/CTNND1 axis in vitro and in vivo.

CTNND1 is a member of the cadherin-catenin complex that regulates the Wnt/β-catenin signaling pathway [37]. Masses of evidence have verified that CTNND1 regulates the occurrence and development of tumors [38]. The article of Tang stated CTNND1 could elevate proliferation, migration, and invasion of hepatoma cells in vitro. It could also promote tumor formation and metastasis of mouse liver cancer cells [39–41]. An earlier paper exhibited that CTNND1 knockdown blocked proliferation, migration, and invasion in CRC cells [42]. Consistent with these opinions, our results suggested that CTNND1 expression was robustly up-regulated in CRC tissues and cells, and suppressed cell proliferation, migration, invasion and triggered apoptosis in SW480 and LS1034 cells. Moreover, CTNND1 overexpression exhibited an EMT phenotype and up-regulated the levels of CyclinD1 and MMP9. The level of Cleaved-casp-3 protein, a symbol of apoptosis, was decreased, and Bcl-2 protein was increased. As we all know, β-catenin involves in the regulation of CyclinD1 and MMP9 in tumor cells [43]. So CTNND1 regulates the growth of CRC cells by inactivating the apoptosis pathway and activating part of Wnt/β-catenin signals. As a more specific mechanism has yet to be explained, we will continue to explore the downstream molecular mechanism of the KCNQ1OT1/miR-329-3p/CTNND1 axis in the subsequent study.

## Conclusions

**Fig. 9:**
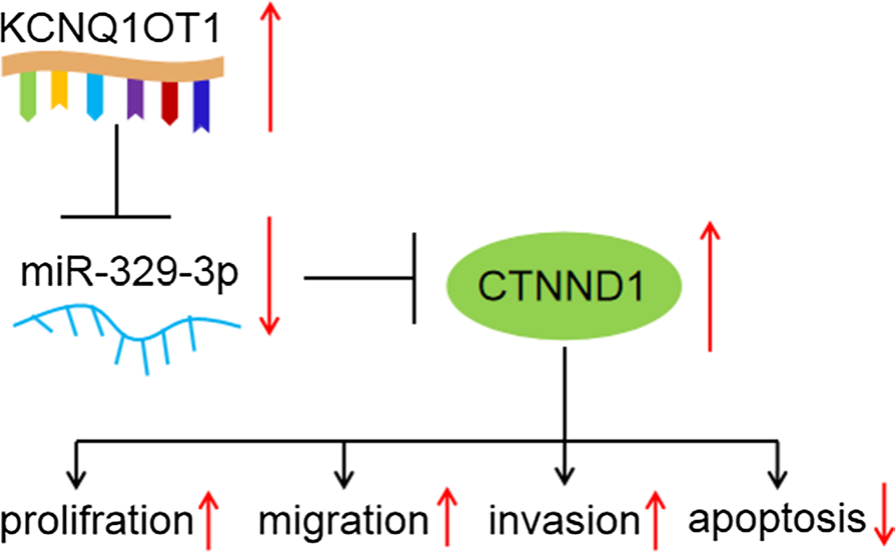
Schematic diagram showing the effect of the KCNQ1OT1/miR-329-3p/CTNND1 on proliferation, migration, invasion, and apoptosis in CRC cells

Our research demonstrated that KCNQ1OT1 induced proliferation, migration, invasion, and restrained apoptosis through up-regulating CTNND1 mediated by miR-329-3p in CRC (Fig. 9), which may conduce to novel insights for CRC therapy. Nevertheless, plenty of clinical samples are needed to elucidate the exact molecular mechanisms/activity of the KCNQ1OT1/miR-329-3p/CTNND1 axis during the progression of CRC and to further explore other possible targets of KCNQ1OT1 in CRC.

## Supplementary information

**Additional file 1: Figure S1.** The survival rate diagram of KCNQ1OT1 in TCGA database was shown.

## Data Availability

All data generated or analyzed during this study are included in this published Article.
