# Supplementary figures and images for: Long non-coding RNA KCNQ1OT1 up-regulates CTNND1 by sponging miR-329-3p to induce the proliferation, migration, invasion, and inhibit apoptosis of colorectal cancer cells
Source: Cancer Cell Int. 2020 Jul 24;20:340. doi: 10.1186/s12935-020-01425-2 (PMC7379774; doi:10.1186/s12935-020-01425-2)

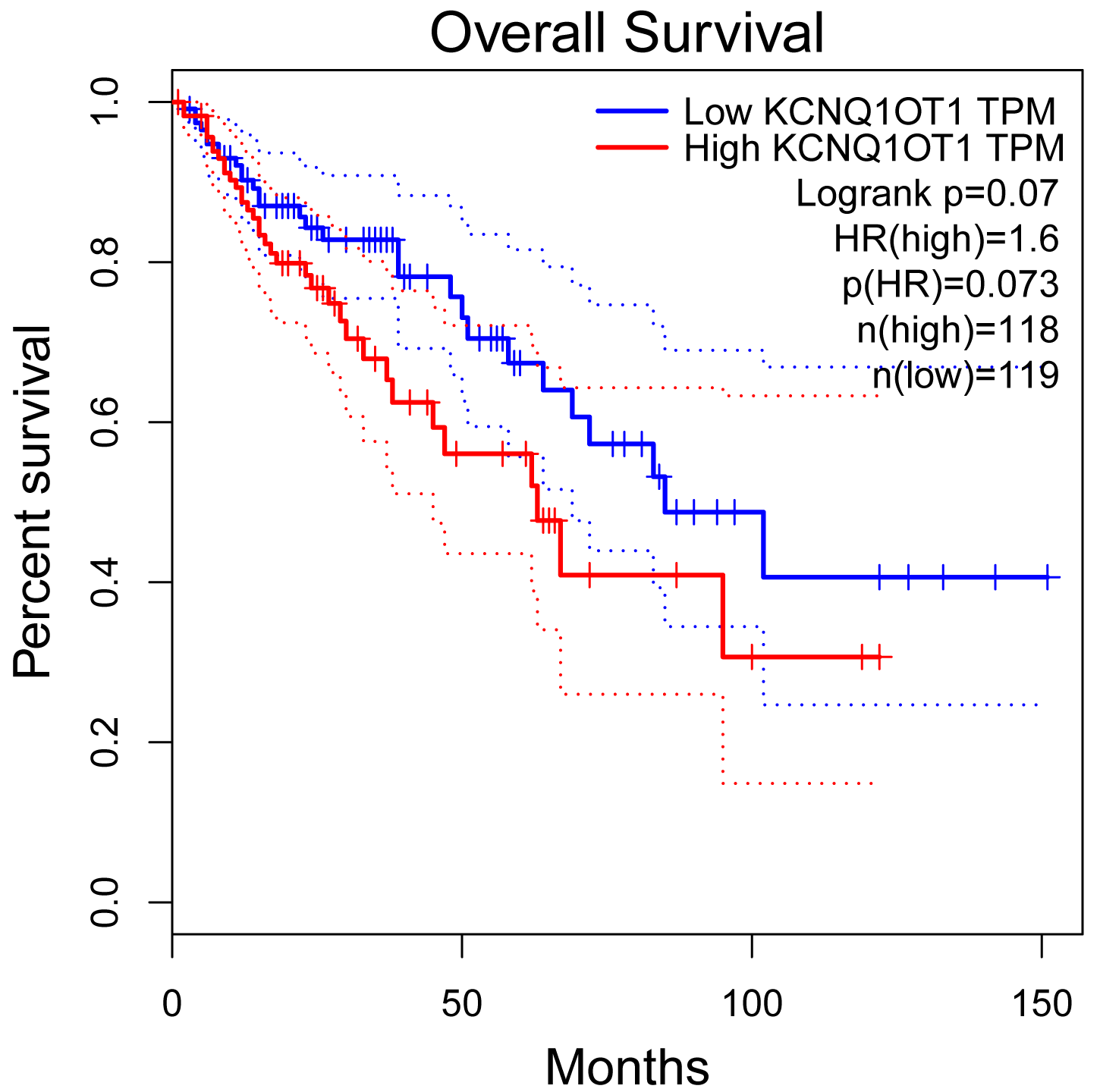

Supplement: Supplementary file 1 — Additional file 1: Figure S1. The survival rate diagram of KCNQ1OT1 in TCGA database was shown. [file 12935_2020_1425_MOESM1_ESM.tif]
